# Correlation of cardiac magnetic resonance imaging and electrophysiology study findings among patients with frequent premature ventricular contractions

**DOI:** 10.1186/1532-429X-14-S1-O104

**Published:** 2012-02-01

**Authors:** Adam Helms, Hutsaya Prasitdumrong, Prachi Agarwal, Gisela C Mueller, Frank Bogun

**Affiliations:** 1 University of Michigan, Ann Arbor, MI, USA

## Summary

Despite inclusion in the diagnostic criteria for arrhythmogenic right ventricular cardiomyopathy, this study of 87 consecutive patients with isolated frequent LBBB-morphology PVCs and high PVC burden (mean 19%) demonstrated only a rare association with CMR diagnostic criteria for ARVC, no cases of ARVC diagnosis, no association of minor wall motion abnormalities (hypokinesis) with markers of ARVC, and no cardiac events in follow-up. Interestingly, however, RV dilation was very common and paralleled LV dilation.

## Background

Frequent premature ventricular contractractions (PVCs) is typically a benign disorder, but has become a common indication for cardiac magnetic resonance imaging (CMR) due to inclusion in diagnostic criteria for arrhythmogenic right ventricular cardiomyopathy (ARVC). A recent study also suggested a worse prognosis in these patients associated with findings on CMR. The purpose of this study was to correlate CMR data with electrophysiology study (EP) findings and clinical outcomes in patients with frequent PVCs.

## Methods

We studied 87 consecutive patients referred for catheter ablation of isolated frequent left bundle branch block morphology PVCs. The 2010 ARVC Task Force criteria were used to define major and minor criteria for ARVC. The CMR exam included steady-state free precession imaging for wall motion and volume/function quantification and black blood imaging for fatty infiltration. Major RV wall motion abnormalities (WMA) were defined by akinesis, dyskinesis, or dyssynchrony, while minor wall motion abnormalities were defined by hypokinesis. The EP study included program stimulation for inducibility of VT and either activation or pace mapping for determination of PVC origin.

## Results

The mean PVC burden was 19 + 13%, and no patients had a history of syncope or family history of early sudden death. 15 patients met a minor (10) or major (5) Task Force criterion for ARVC by EKG. Major WMAs by CMR were present in 2 (2%) of patients, but only 1 of these had RV dilation and the PVCs in this case mapped to the left ventricle. No patients met full diagnostic criteria for ARVC. Hypokinetic segments were present in 7 patients, but did not correlate with EKG criteria for ARVC (p=0.41), NSVT on 24-hour EKG (p=0.51), RV dilation (p=0.43), RV fatty infiltration (p=1.0), inducibility of VT (p=1.0), or RV versus LV site of origin of the PVC (p=0.67). RV dilation was present in 74 (84%) patients, and correlated with LV dilation (R^2^=0.69, see Figure). RV dysfunction was present in 34 (39%). During a median follow-up of 16 (range, 3 - 71) months, no syncope, VT, heart failure, or sudden death was documented.

**Figure 1:**
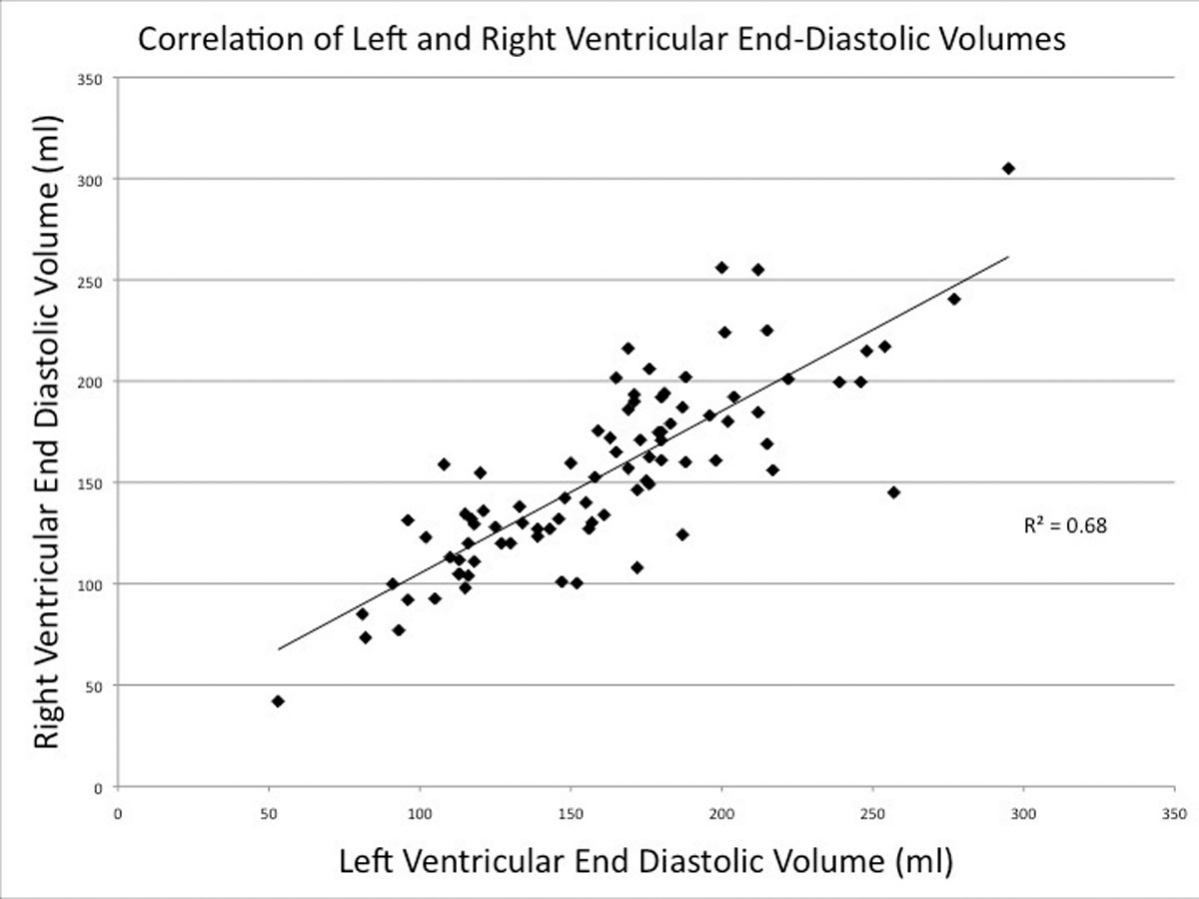


## Conclusions

Despite frequent RV dilation that paralleled LV dilation, other CMR findings diagnostic of ARVC in patients with isolated LBBB-morphology PVCs are rare. Hypokinetic segments do not correlate with EKG criteria for ARVC, NSVT, RV dilation, fatty infiltration, inducible VT, or site of origin of the PVC. Frequent PVCs were associated with a benign clinical course, regardless of the presence of RV dilation or hypokinetic segments.

## Funding

None.

